# mTOR inhibition enhances efficacy of dasatinib in *ABL*-rearranged Ph-like B-ALL

**DOI:** 10.18632/oncotarget.24020

**Published:** 2018-01-06

**Authors:** Moran Gotesman, Thanh-Trang T. Vo, Lee-Or Herzog, Tiffeny Tea, Sharmila Mallya, Sarah K. Tasian, Marina Konopleva, David A. Fruman

**Affiliations:** ^1^ Department of Molecular Biology and Biochemistry, University of California, Irvine, CA, USA; ^2^ Hyundai Cancer Institute, CHOC Children’s Hospital, Orange, CA, USA; ^3^ Department of Pediatrics, Division of Oncology, Center for Childhood Cancer Research, Children's Hospital of Philadelphia and Perelman School of Medicine at the University of Pennsylvania, Philadelphia, PA, USA; ^4^ Department of Leukemia, The University of Texas MD Anderson Cancer Center, Houston, TX, USA

**Keywords:** acute leukemia, animal model, childhood leukemia, leukemia therapy, tyrosine kinases

## Abstract

High-risk subtypes of B-cell acute lymphoblastic leukemia (B-ALL) include Philadelphia chromosome-positive (Ph+) B-ALL driven by the *BCR-ABL1* oncogene and a more recently identified subtype known as *BCR-ABL*-like or Ph-like B-ALL. A hallmark of both Ph+ and Ph-like B-ALL is constitutive activation of tyrosine kinase signaling that is potentially targetable with tyrosine kinase inhibitors (TKIs). B-ALL cells also receive extracellular signals from the microenvironment that can maintain proliferation and survival following treatment with TKIs. Therefore, there is strong rationale for combining TKIs with other therapies targeting signal transduction pathways. Here we show that combinations of the ABL-directed TKI dasatinib with mTOR kinase inhibitors (TOR-KIs) are more effective than TKI alone against patient-derived Ph-like B-ALL cells harboring rearrangements of *ABL1* or *ABL2*. We also report the establishment of a new human Ph-like B-ALL cell line that is stromal cell-independent *in vitro* and can be used for xenograft experiments *in vivo*. These findings provide rationale for clinical testing of TKI plus TOR-KIs in children and adults with Ph-like B-ALL and a new experimental tool to test promising therapeutic strategies in this poor prognosis subtype of B-ALL.

## INTRODUCTION

B-lymphoblastic leukemia (B-ALL) is the most common pediatric cancer. While event-free survival exceeds 85% for most children treated with contemporary therapy, outcomes are very poor for patients who relapse, highlighting a need for further research and new treatments. In particular, children, adolescents, and adults with Philadelphia chromosome-like (Ph-like) B-ALL have high rates of relapse and mortality with conventional chemotherapy [1–5]. This leukemia subtype lacks *BCR-ABL1* rearrangement, but has similar activated tyrosine kinase signaling and gene expression profiles as those of Ph+ B-ALL [6]. Approximately half of Ph-like B-ALL patients have *CRLF2* (*cytokine receptor-like factor 2*) overexpression, with or without accompanying mutations in JAK2 tyrosine kinase, and are candidates for treatment with JAK inhibitors [6, 7]. Another subset of Ph-like leukemia have ‘ABL class’ fusions involving *ABL1*, *ABL2*, *CSF1R*, and *PDGFRB* that are amenable to SRC/ABL tyrosine kinase inhibitors (TKIs) such as imatinib and dasatinib [6, 7]. Currently patients with NCI high-risk B-ALL treated on Children’s Oncology Group (COG) protocols are being tested in real-time for Ph-like ALL-associated alterations and are eligible to participate in clinical trials incorporating dasatinib or ruxolitinib with post-induction chemotherapy (NCT01406756 and NCT02723994) [7]. However, it is plausible that patients with Ph-like ALL may develop resistance to specific targeted therapies, similar to TKI resistance seen in patients with Ph+ B-ALL, and thus alternative therapeutic strategies should be explored. We hypothesized that addition of a mammalian target of rapamycin (mTOR) kinase inhibitor (TOR-KI) could prevent this resistance and further decrease overall leukemia burden, as TOR-KIs suppress proliferation and survival signals downstream of both the oncogene and extracellular inputs [8]. We previously tested this combination in models of Ph+ B-ALL and found greater anti-leukemia effects when dasatinib was combined with TOR-KI compounds PP242 or MLN0128 [9, 10]. Other groups reported similar findings using other TOR-KIs, such as OSI-027 [11]. Based on these results, we reasoned that dual kinase inhibitor (TKI plus TOR-KI) therapy would similarly provide superior anti-leukemia cytotoxicity in patient-derived xenograft (PDX) models of childhood Ph-like B-ALL. In this study, we show that the anti-leukemia effect of dasatinib is enhanced by the TOR-KIs compound AZD2014 at doses that do not fully block mTOR activity as a single agent and preserve normal bone marrow cell proliferation. To accelerate further studies of *ABL*-rearranged Ph-like B-ALL, we also describe a novel stroma-independent Ph-like ALL cell line from a child with a somatic *ETV6-ABL1* translocation that is suitable for *in vitro* and *in vivo* studies.

## RESULTS

### A TOR-KI enhances anti-leukemia efficacy of dasatinib *in vivo*

**Figure 1:**
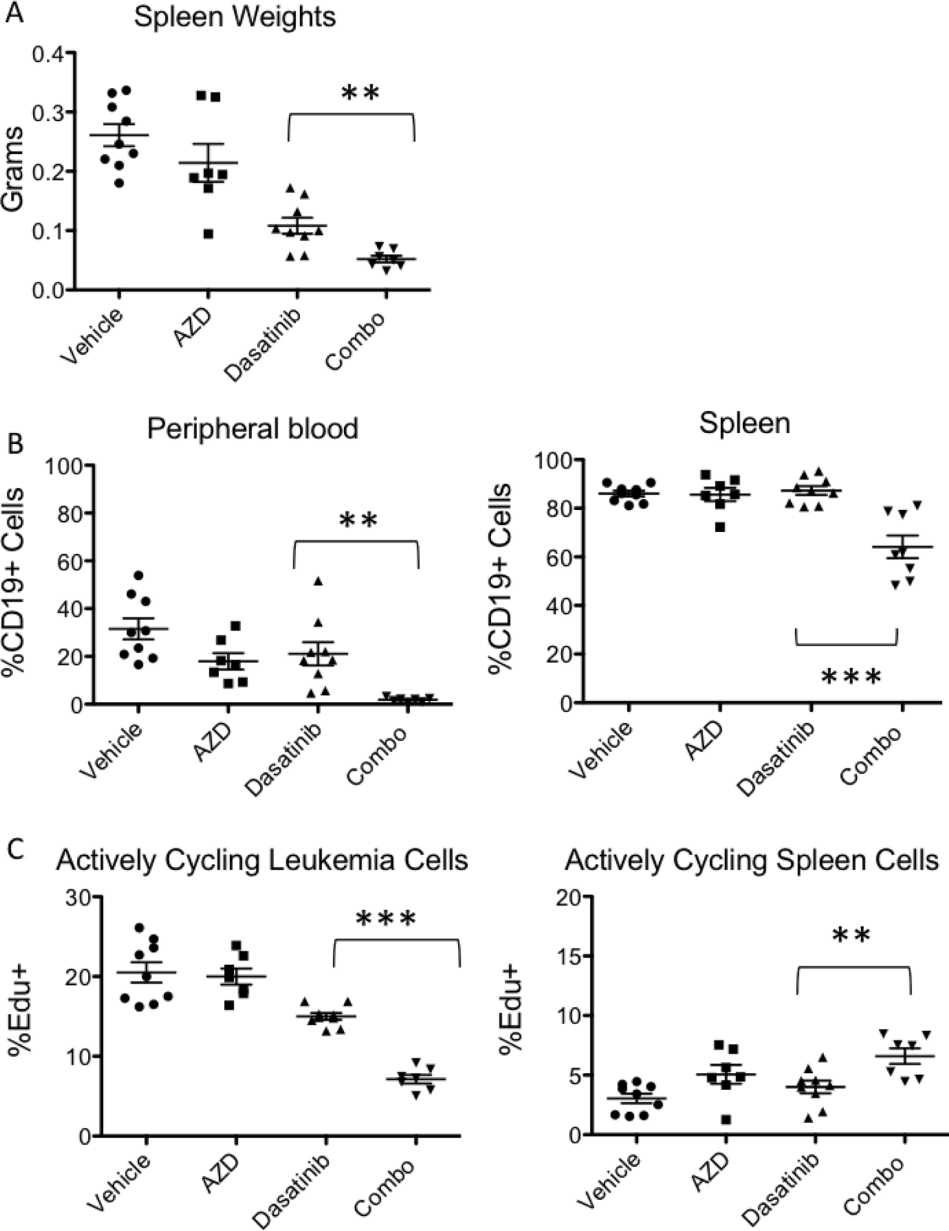
Combination TKI and TOR-KI treatment decreases leukemia burden *in vivo* A PDX model of Ph-like B-ALL (PAUXZX) was treated with dasatinib (2.5 mg/kg), AZD2014 (20 mg/kg), or both (abbreviated “combo”) daily for 8 days. (**A**) Decreased spleen size in combination group as compared to either single treatment group and significantly decreased spleen weight in the combination group when compared to dasatinib group (*p* = 0.0038). (**B**) Leukemia burden was assessed by percent hCD19+ cells in the spleen and peripheral blood by flow cytometry. Combination treatment significantly reduced ALL burden compared to single-agent dasatinib (*p* = 0.0002 and *p* = 0.0038 by two-tailed unpaired *t*-test (**C**) Actively cycling cells were identified by the incorporation of EdU in human leukemia cells and murine spleen cells. A significant decrease in actively cycling hCD19+ ALL cells was detected in the combination group versus the dasatinib monotherapy group (*p* < 0.0001). There was a significant increase in actively cycling murine (non-leukemia) spleen cells identified by mCD45 in the combination group as compared to the dasatinib group (*p* = 0.0082 by two-tailed unpaired *t*-test).

We tested the combination effect of the TKI dasatinib with the TOR-KI AZD2014 in a murine PDX model of childhood Ph-like B-ALL harboring an *ETV6-ABL1* fusion. For these studies, we used a dose of dasatinib (2.5 mg/kg via oral gavage once daily) that reduces, but does not completely eliminate leukemia *in vivo*. We used AZD2014 at a dose (20 mg/kg via oral gavage once daily) previously shown to reduce growth of solid tumors *in vivo* in xenograft mouse models [12]. After 8 days of oral dosing with vehicle, AZD2014, dasatinib, or the combination, mice were sacrificed, and spleen and bone marrow were analyzed for tumor burden. Spleen size and weight were significantly decreased in the combination group as compared to the group treated with dasatinib monotherapy (Figure 1A). There was also a significant decrease in leukemia burden as assessed by percent human (h) CD19+ cells within the peripheral blood and spleen in the combination treated group versus dasatinib alone (Figure 1B), which are concordant with our recent study testing various PI3K pathway inhibitors in other Ph-like B-ALL xenografts [13]. To test for the selectivity of the treatments for leukemia cells, we analyzed cycling cells within subpopulations by measuring EdU incorporation (Figure 1C). There was a significant reduction of cycling cells in the leukemia (hCD19+) populations within the spleen when comparing combination treatment to dasatinib monotherapy. In contrast, combination treatment enhanced cycling of endogenous murine CD45+ leukocytes (Figure 1C), likely due to diminished competition with leukemia cells. Similar results were observed in a second animal study in which PAUXZX ALL PDX mice were treated with the structurally related TOR-KI AZD8055 (Supplementary Figure 1).

**Figure 2:**
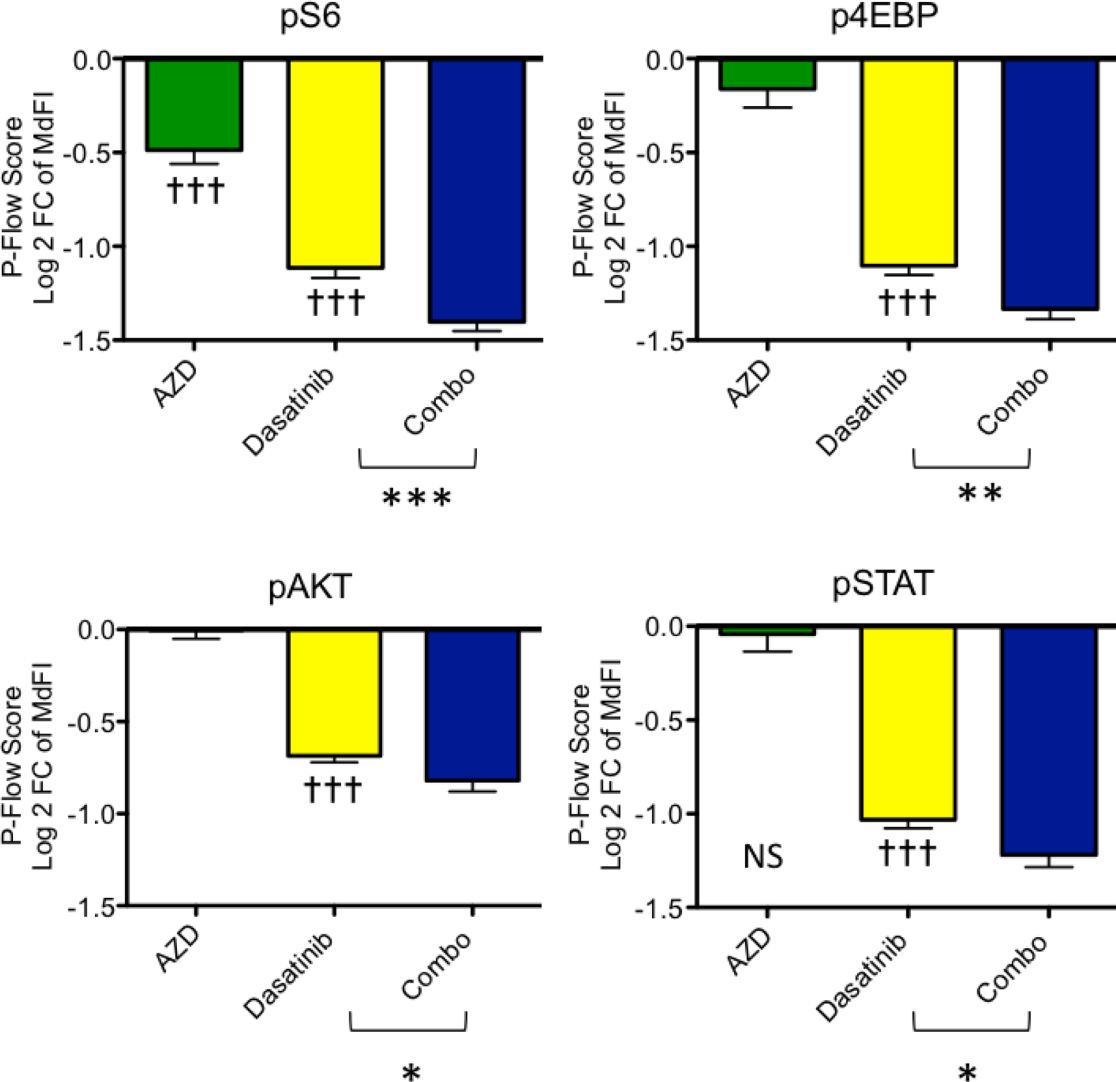
Pharmacodynamic analysis of Ph-like B-ALL cells after *in vivo* treatment mTORC1 and mTORC2 readouts were analyzed by phosphoflow cytometry in bone marrow cells isolated from ALL PDX mice in Figure 1. Signaling changes are shown as a p-flow score, defined as the log_2_ of ratio of median fluorescence intensities of the treated sample relative to the untreated. Decreased phosphorylation results in a negative p-flow score. The untreated sample has a p-flow score of zero and is not displayed graphically. Dasatinib reduced pSTAT5 and mTOR readouts pS6, p4E-BP1 and pAKT (^†††^*p* < 0.0001, one-sample *t*-test compared to zero). In PDX animals treated with AZD2014 at 20 mg/kg once daily for 8 days, only pS6 was significantly inhibited (^†††^*p* < 0.0001, one-sample *t*-test compared to zero). The combination of AZD2014 with dasatinib significantly reduced all signaling readouts versus dasatinib monotherapy (^***^p = 0.0009 for pS6; ^**^p = 0.004 for p4E-BP1, ^*^p = 0.03 for pAKT, *p* = 0.01 for pSTAT; unpaired one-tailed *t*-tests).

Phosphoflow measurement of signaling was used to assess the pharmacodynamic activities of dasatinib, AZD2014, and combination treatment in the *in vivo* experiment shown in Figure 1. Mice were euthanized after 8 days of treatment, and cell samples were obtained 2 hours after the last inhibitor dose. Interestingly, AZD2014 at 20 mg/kg dosing only partially reduced pS6 and had no significant effect on p4E-BP1 (both of these are downstream readouts of mTORC1 activity) or pAKT^S473^ (a readout of mTORC2 activity) (Figure 2 and Supplementary Figure 2). These results demonstrate that the given dose of AZD2014 caused incomplete target inhibition. Conversely, dasatinib strongly suppressed pSTAT5, confirming complete inhibition of proximal ABL-mediated signaling. Similarly, dasatinib treatment significantly, but incompletely, reduced pS6, p4E-BP1 and pAKT. Notably, the combination of dasatinib with AZD2014 caused significantly greater inhibition of both mTORC1 and mTORC2 readouts compared to either single agent (Figure 2).

### TKI plus TOR-KI combination enhances cytotoxicity *in vitro*

**Figure 3:**
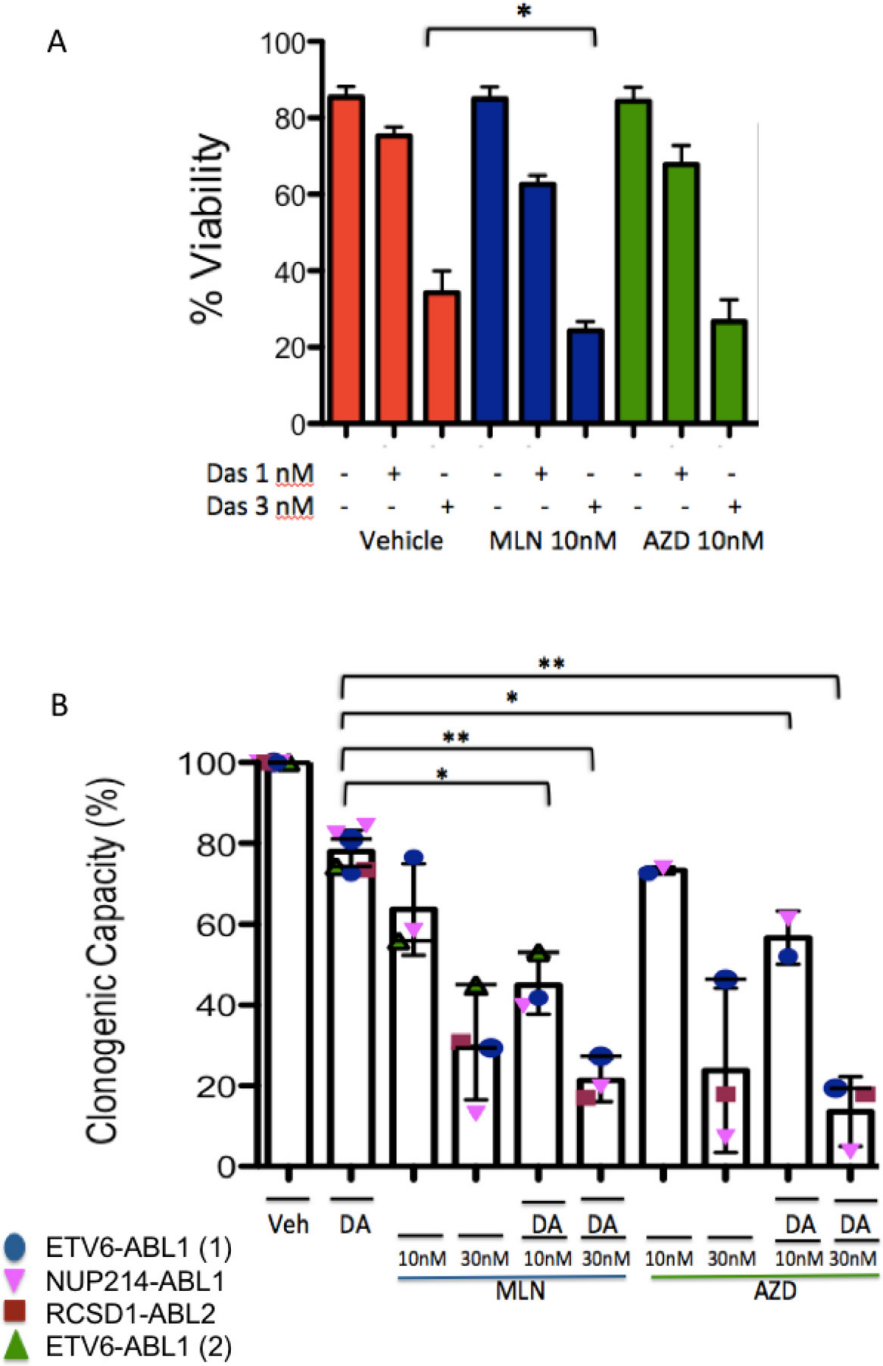
Effects of single and combination treatments on survival and colony formation *in vitro* (**A**) PAUXZX ALL PDX cells were grown *in vitro* on stroma and treated with two different TOR-KI compounds (MLN0128 and AZD2014) in combination with two concentrations of TKI (dasatinib 1 nM and 3 nM). A significant combination effect was seen with dasatinib 3 nM and MLN0128 10 nM (*p* = 0.02, two-tailed paired *t* test). (**B**) Four *ABL1-* or *ABL2*-rearranged Ph-like ALL patient samples (Table 1) were tested in colony assays with dasatinib (1 nM) in combination with MLN0128 and AZD2014 at 10 nM and 30 nM. There was a significant reduction when dasatinib was combined with TOR-KIs (MLN1028 *p* = 0.02, *p* = 0.007, AZD2014 *p* = 0.04, 0.004, two-tailed paired *t* test).

To explore further the combination effect of TKI with TOR-KI, we performed additional *in vitro* analyses using PDX cells. We tested two different TOR-KI compounds, MLN0128 and AZD2014. The combination of dasatinib (3 nM) with MLN0128 (10 nM) reduced viability significantly more than dasatinib alone in *ETV6-ABL1* cells cultured on stroma cells (Figure 3A). We observed a similar trend when dasatinib (1 nM) and (3 nM) was combined with AZD2014 (10 nM) (*p* = 0.09 and *p* = 0.18 respectively). Due to low viability of some patient samples when cultured in liquid media on stroma, we performed colony-forming assays in the presence of human cytokines. In assays using 4 different *ABL1*- or *ABL2*-rearranged patient samples (Table 1) including two specimens with *ETV6-ABL1*, co-treatment with 1 nM dasatinib and MLN0128 or AZD2014 at 10 nM or 30 nM reduced colony numbers more than dasatinib alone (Figure 3B).

**Table 1:** Ph-like B-ALL specimens

COG USI^*^	Ph-like *ABL*-class fusion	Age	Sex	Ethnicity	Induction Protocol
PAUXZX	*ETV6-ABL1*	5	F	Caucasian/ Non-Hispanic	AALL1131
PAVMLC	*RCSD1-ABL2*	2	F	Hispanic/Latino	AALL1131
PAKVKK	*NUP214-ABL1*	4	M	Hispanic/Latino	P9906
PANSFD	*ETV6-ABL1*	5	M	Caucasian/ Non-Hispanic	AALL0232

^*^COG USI = Children’s Oncology Group Unique Specimen Identifier.

To determine the effect of inhibitors on mTOR activity in stroma-supported cultures of *ETV6-ABL1* cells, we used phosphoflow cytometry to measure the mTORC1 readout pS6 (Supplementary Figure 3). As expected, dasatinib and both TOR-KIs compounds partially decreased pS6 with greater effects seen in the combination group. This observation was consistent with PD data from PDX mice treated *in vivo*. However, basal signaling was consistently low in B-ALL cells cultured on stroma, a condition that maintains B-ALL viability but does not support proliferation. This low basal signaling reduced the dynamic range of signaling measurements.

### Ph-like B-ALL cell line

**Figure 4:**
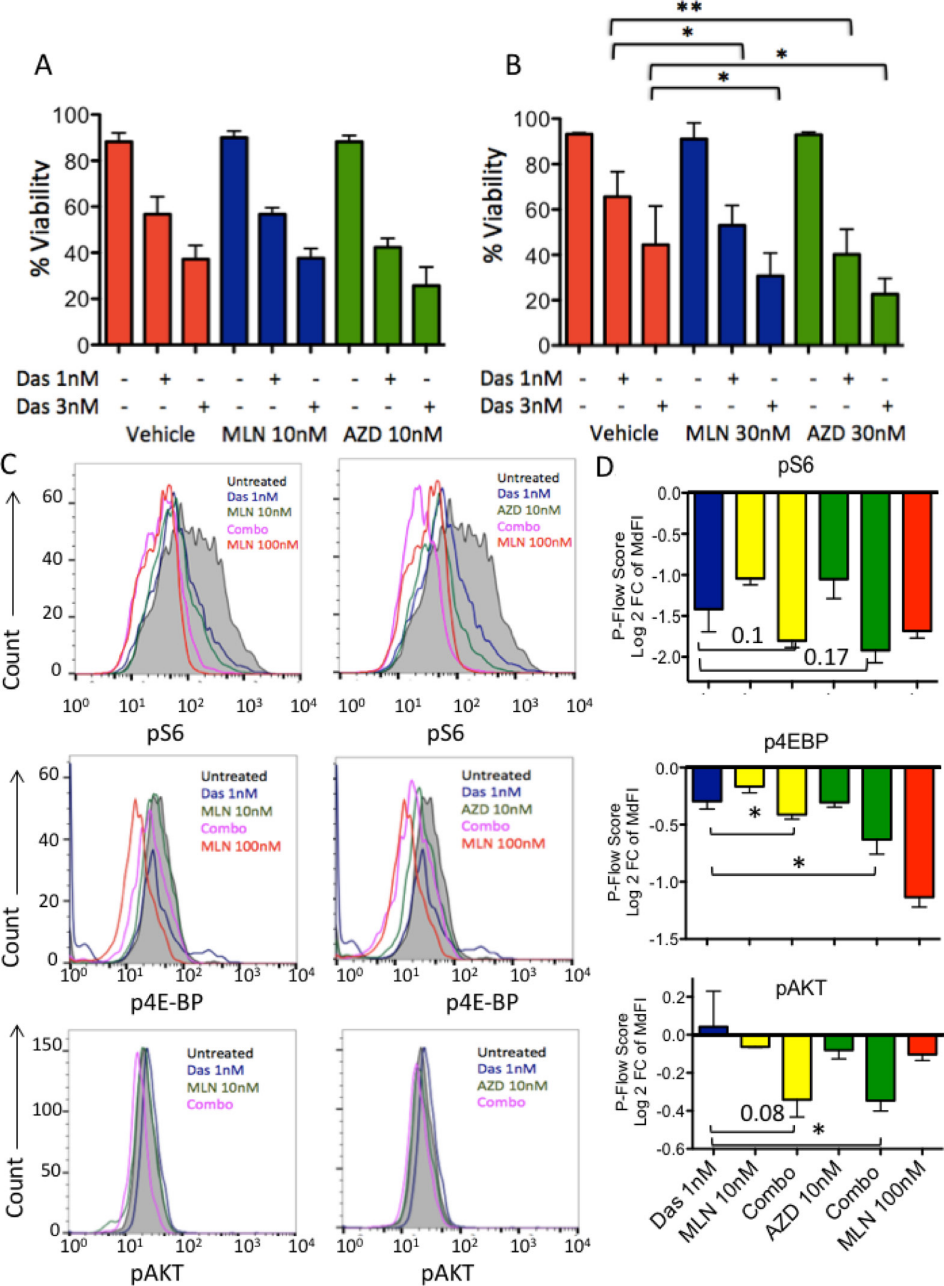
*In vitro* survival and signaling inhibition of the TVA1 stroma-independent Ph-like ALL cell line with *ETV6-ABL1* rearrangement TVA1 cells in suspension culture were treated with dasatinib 1 nM or 3 nM in combination with MLN0128 and AZD2014 10 nM and 30 nM (**A**) A significantly greater effect was observed in cells treated with the combination of dasatinib 1 nM and 3 nM with MLN0128 and AZD2014 at 30 nM compared to dasatinib alone (*p* = 0.004, 0.04 and < 0.001, 0.01, two-tailed paired *t* test). (**B**–**D**) Phosphoflow readouts for mTORC1 (pS6 and p4E-BP1) and mTORC2 (pAKT). Incomplete reduction in phosphorylation with single treatment with either TKI or TOR-KI was observed, but more complete inhibition of phosphorylation was achieved in the combination treatment groups.

During the process of analyzing combination effects of TKI and TOR-KI compounds on expanded B-ALL cells, we created a stromal cell-independent Ph-like cell line (termed TVA1) from a PDX model derived from a bone marrow specimen from a 5-year old female with Ph-like ALL with an *ETV6-ABL1* fusion (PAUXZX) treated on the Children’s Oncology Group protocol AALL1131. Primary cells were first cultured on OP9 stroma and maintained in the same medium (Alpha-MEM, 20% FBS, sodium-pyruvate, glutamax and penicillin/streptomycin) without stroma at 1 million cells/mL and passaged every 3–4 days. After over 20 passages, the authenticity of this cell line compared to the original sample was verified by short tandem repeat testing (data not shown). TVA1 cells expressed typical B-ALL markers (CD10^+^CD19^+^CD20^+^; Supplementary Figure 4), proliferated without growth factors or stromal support, and retained *in vitro* sensitivity to dasatinib in cytotoxicity assays (Figure 4A–4B) and high basal pSTAT5 by phosphoflow cytometry analysis (data not shown). When dasatinib (1 nM and 3 nM) was combined with MLN0128 (10 nM) or AZD2014 (10 nM), there was no significant difference in viability compared with dasatinib alone. However, at higher concentrations of MLN0128 (30 nM) or AZD2014 (30 nM), we observed a significant decrease in viability in combination treatments with dasatinib (1 nM and 3 nM) compared to each of the drug treatments alone. In phosphoflow cytometry assays, the TVA1 cell line showed similar patterns as observed in the original stromal-dependent *ETV6-ABL1* sample. Specifically, single treatment with either TKI or mTOR inhibitor caused an incomplete reduction of mTORC1 readouts pS6 and p4E-BP, whereas more complete inhibition occurred when the two drugs were combined (Figure 4C–4D). Phosphoflow assessment of the mTORC2 readout pAKT in this cell line had a narrow dynamic range, but showed a similar trend towards greater inhibition by the combination treatments (Figure 4C–4D).

**Figure 5:**
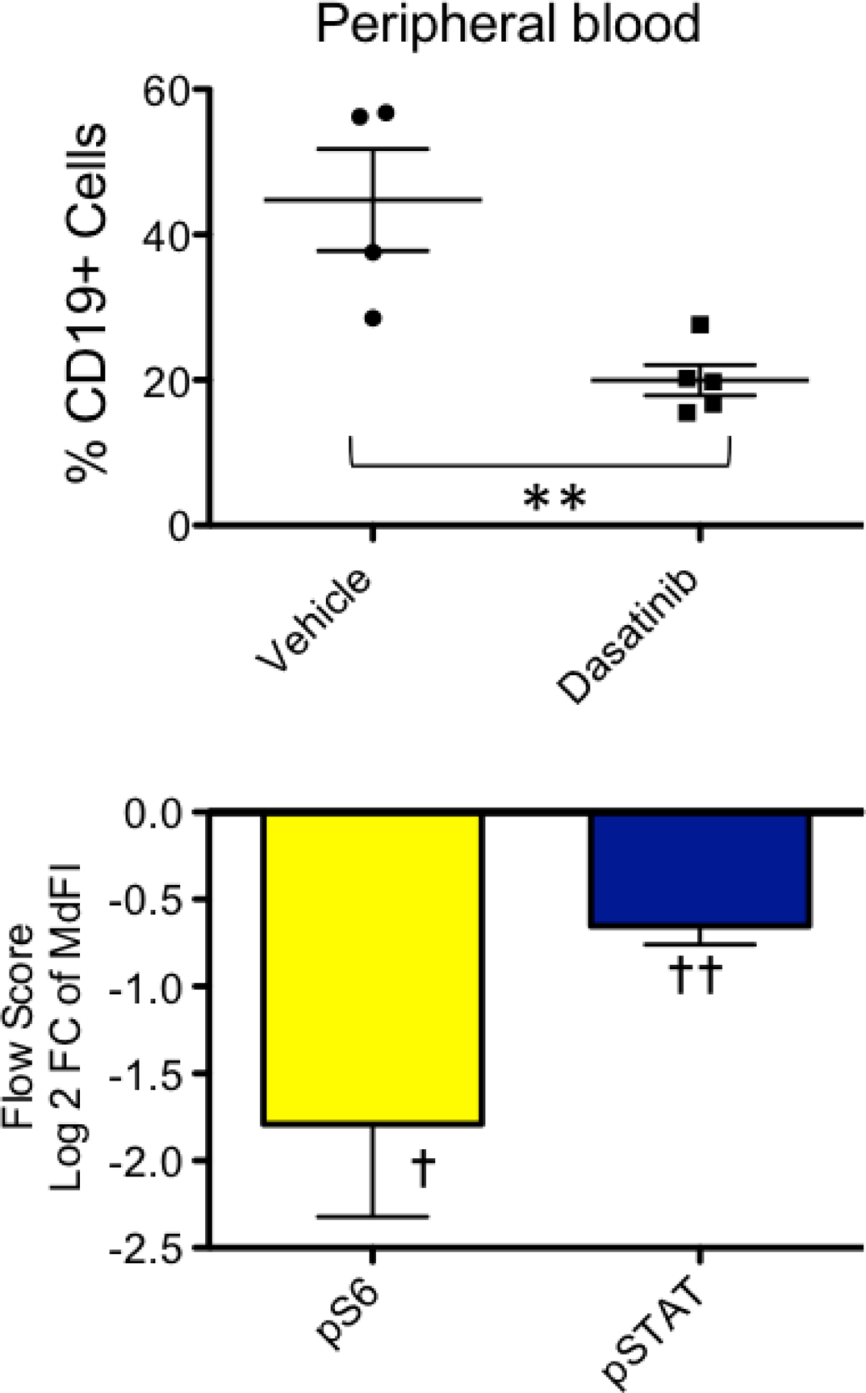
Confirmation of *in vivo* engraftment and dasatinib sensitivity of Ph-like ALL TVA1 cells Immunodeficient NSG mice were injected with TVA1 cells, and peripheral blood engraftment of ALL was documented at 3 weeks. Mice were then treated for 5 days with dasatinib 5 mg/kg by oral gavage once daily. Leukemia burden in the peripheral blood was significantly reduced post-treatment, as assessed by flow cytometric quantification of percent hCD19 cells (^**^p = 0.007, unpaired *t*-test). Phosphoflow cytometry analysis of gated human B-ALL cells in blood and spleen demonstrated significant inhibition in pS6 and pSTAT5 levels (one-sample *t*-test compared to zero, ^†^*p* = 0.03 and ^††^*p* = 0.003, respectively).

We also explored the potential of TVA1 to engraft in immunodeficient mice. Two million TVA1 cells were injected i.v. into NSG mice with demonstrated engraftment in peripheral blood after 2–3 weeks. We then treated PDX mice for 5 days with dasatinib 5 mg/kg via oral gavage once daily and confirmed a significant decrease in leukemia burden compared to vehicle-treated mice after five days of treatment (Figure 5). As expected, dasatinib treatment of TVA1 PDX mice also decreased pS6 and pSTAT in gated leukemia cells in pharmacodynamic assays conducted at the end of the treatment period (Figure 5).

## DISCUSSION

Adults and children with Ph-like ALL have inferior outcomes with conventional chemotherapy treatment, and it is imperative to develop more effective therapies for these patients [1–5]. Approximately 10–15% of children, adolescents, and young adults with Ph-like ALL have ABL class alterations (*ABL1*, *ABL2*, *CSF1R,* or *PDGFRB*) that are targetable with imatinib or dasatinib [6, 7]. Results from our current study are concordant with the recently reported combination effect of TKIs with mTOR inhibitors to enhance anti-leukemia potential [13]. That study tested several selective PI3K pathway-targeting drugs, including isoform-selective PI3K inhibitors (BYL719, idelalisib), a dual PI3K/mTOR inhibitor (gedatolisib), and the TOR-KI AZD2014. While many pan-PI3K or PI3K/mTOR inhibitors have demonstrated poor clinical safety profiles [14], several recent phase I clinical trials testing TOR-KIs compounds have reported acceptable safety and preliminary activity in patients with relapsed/refractory cancers [15–17]. One of these compounds (MLN0128) is also currently in phase 2 clinical testing in patients with relapsed ALL (NCT02484430). Interestingly, enhanced mTOR inhibition was observed in our PDX model treated with subtherapeutic dosing of the TOR-KI AZD2014 in combination with dasatinib, suggesting a potential therapeutic window for combination inhibitor strategies. This is an important finding since some mTOR inhibitors have been very poorly tolerated when combined with intensive chemotherapy generally required to achieve cure [18]. These findings may suggest there is an ability to use submaximal dosing when mTOR inhibitors will be combined with other targeted therapies, such as TKIs, that indirectly reduce mTOR signaling. This combination effect is promising and should be explored in the clinic.

Careful biochemical and mechanistic characterization of aberrant signaling in Ph-like ALL is critical for improving understanding of the biology and therapeutic options for patients with these high-risk leukemias. While several human cell lines exist for the most common *CRLF2*-rearranged Ph-like ALL subtype (MUTZ5, MHH-CALL-4, and INC) [19], there are no known Ph-like ALL cell lines harboring ABL-class alterations. In the current study, we created a robust new *ABL1*-rearranged Ph-like cell line, TVA1, that grows reproducibly *in vitro* without stroma support requirement and is engraftable in NSG mice for *in vivo* experimental studies.

In summary, our findings provide new insights about therapeutic approaches in Ph-like B-ALL with ABL-class alterations and a new cellular tool with which to study the biology of this high-risk leukemia subtype. Given their relative rarity, the ABL-class Ph-like ALL subtype has been less well-characterized compared to more common *CRLF2*-rearranged ALLs. Our data support further exploration of dual kinase inhibitor (TOR-KI/TKI) approaches in ABL-class Ph-like B-ALL. Such combinations have potential to enhance efficacy without increasing toxicity and may minimize potential for compensatory signaling upregulation that can lead to therapeutic resistance.

## MATERIALS AND METHODS

### Cell culture and patient samples

Bone marrow specimens from children with high-risk B-ALL (by National Cancer Institute-Rome criteria) were originally obtained via informed consent on approved COG protocols P9906, AALL08B1, and AALL1131 in accordance with the Declaration of Helsinki. These leukemias were then established as patient-derived xenograft (PDX) models in immunocompromised mice as described [4, 20] and stored as coded samples without identifying patient health information in biobanks by the COG and the Children’s Hospital of Philadelphia (Table 1). Approval for B-ALL PDX sample use was obtained via COG biology protocols AALL12B13-Q and AALL17B6-Q. These studies were considered non-human subjects research by the Institutional Review Board of the University of California, Irvine.

Xenografted human leukemia cells from murine spleens were cultured on irradiated OP9 stromal cell layers and maintained in primary media including MEM Alpha, 20% fetal bovine serum (FBS), 1 mM sodium pyruvate, 1X GlutaMAX and 100 U/mL penicillin/streptomycin. T25 flasks were initially coated with 1% gelatin to help with OP9 adherence. OP9 stromal cells were irradiated at 2500 Rad and plated onto flasks one day prior to plating PDX leukemia cells. Cells were maintained in a 37°C incubator with 5% CO_2_ and replated with fresh media and OP9 cells every 5 days. The immortalized stromal cell-independent cell line TVA1 was established by these methods from a Ph-like ALL specimen with *ETV6-ABL1* fusion (PAUXZX; Table 1).

### Reagents

AZD2014, MLN0128 and dasatinib were purchased from LC laboratories. Each inhibitor was dissolved in DMSO to the final concentration of 1 mM and stored at –80°C.

### Assessment of cell apoptosis (cell death assays)

ALL cells were incubated with dasatinib, MLN0128, AZD2104 or combinations for 72 hours in 200 mL of primary media per well in 96-well flat bottom plates. Irradiated OP9 cells were plated the day prior on gelatin pre-coated 96-well plates. After incubation, cells were pelleted and resuspended in 150 μL of Annexin Binding Buffer (10 mM HEPES, 140 mM NaCl and 2.5 mM CaCl_2_-2H_2_O, pH 7.4) containing Annexin-V Alexa Fluor 647 and 0.4 μg/mL propidium iodide. Cells were analyzed using a FACSCalibur flow cytometer (Becton Dickinson). Data were processed with FlowJo Software v10.0.8 (FlowJo, LLC) and graphed with Prism (GraphPad).

### Phosphoflow cytometry

ALL cells were incubated with inhibitors for 2 hours on 24-well plates pre-coated with gelatin and irradiated OP9 stromal cells. After incubation, cells were pelleted and incubated in 100 μL 4% paraformaldehyde for 10 minutes at 37°C. Cells were washed with phosphate-buffered saline (PBS), resuspended in 500 μL of 90% ice-cold methanol, and stored at –20°C overnight. The following day, cells were washed with PBS and stained with fluorophore-coupled phospho-specific antibodies (pS6 (S240/244)-AlexaFluor-647, p4E-BP-1 (T37/46)-AlexaFluor-488 or pSTAT5 (Y694)-AlexaFluor- 488, all from Cell Signaling) for 30 minutes. Cells stained with unlabeled anti-pAKT (S473; Cell Signaling) for 30 minutes were then incubated with anti-rabbit AlexaFluor-647 (Thermofisher Scientific). Cells were resuspended in 150 μL PBS and analyzed on a FACSCalibur flow cytometer.

### Colony assays

ALL cells were resuspended in primary media at 2 × 10^5^ cells/condition with recombinant human SCF, IL-7, IL-3, and FLT3 cytokines at 10 ng/mL (Thermo Fisher Scientific). Cells and inhibitors at the designated concentrations were then suspended in Methocult (StemCell Technologies) and vortexed for 90 seconds prior to plating in duplicate in 24-well plates. Colonies were counted twice at 10–14 days after plating by two different people, and number of colonies per condition were averaged for the two counts.

### *In vivo* testing of kinase inhibitors in patient-derived xenograft models

NSG mice were obtained from JAX and bred at UC Irvine. Animal studies were approved by the Institutional Animal Care and Use Committee at UC Irvine. NSG mice at 1–3 months of age were injected retro-orbitally i.v. with 2.5 million ALL cells. Facial vein bleeds were done weekly to monitor engraftment. Upon detection of greater than 1% leukemia in all mice, mice were randomized and treated with 20 mg/kg AZD2014, 2.5 mg/kg dasatinib, or both inhibitors via oral gavage once daily for eight days. AZD2014 was dissolved in 30% Captisol at pH 3, and dasatinib was dissolved in 50:50 mix of polypropylene and water. On day 8, mice were injected with 200 μl of 5 mg/ml EdU intraperitoneally for cell cycle analyses and sacrificed one hour later. Bone marrow, spleen and whole blood were harvested. Red blood cells were lysed in ACK buffer (150 mM NH_4_Cl, 10 mM KHCO_3_, and 0.1 mM Na_2_EDTA in water) for 5 minutes at room temperature and washed with cold PBS + 1% FBS. One million cells were stained with human CD19-PE and mouse CD45-APC for 20 minutes on ice in PBS + 1% FBS. Cells were spun down and resuspended in cold PBS for FACS analysis. The remaining cells were used for phosphoflow analysis and EdU assessment as below.

### EdU detection

Bone marrow and spleen cells were fixed with 4% paraformaldehyde for 10 minutes in room temperature, then washed and stored in PBS and 1% Bovine serum albumin (BSA) in 4 degrees. Cells were washed again in PBS and 1% bovine serum albumin and resuspended in 1X saponin wash buffer for 10 minutes. Cells were washed with saponin buffer and resuspended in Click-iT reaction cocktail (per Life Technologies protocol) for 30 minutes. Cells were then washed with saponin buffer and resuspended in antibody stains for hCD19-PE, mCD45-AlexaFluor 488, and EdU conjugate AlexaFluor 647 (Life Technologies) prior to flow cytometry analysis.

## SUPPLEMENTARY MATERIALS FIGURES



## Abbreviations

B-ALL: B-cell acute lymphoblastic leukemia
Ph+: Philadelphia chromosome-positive
TKIs: tyrosine kinase inhibitors
mTOR: mammalian target of rapamycin
TOR-KIs: mTOR kinase inhibitors
COG: Children’s Oncology Group
PBS: phosphate-buffered saline
FBS: fetal bovine serum
