# Supplementary material for: mTOR inhibition enhances efficacy of dasatinib in ABL-rearranged Ph-like B-ALL
Source: Oncotarget. 2018 Jan 6;9(5):6562–71. doi: 10.18632/oncotarget.24020 (PMC5814232; doi:10.18632/oncotarget.24020)
Supplement: Supplementary file 1 [file oncotarget-09-6562-s001.pdf]

mTOR inhibition enhances efficacy of dasatinib in *ABL*-rearranged Ph-like B-ALL

SUPPLEMENTARY MATERIALS

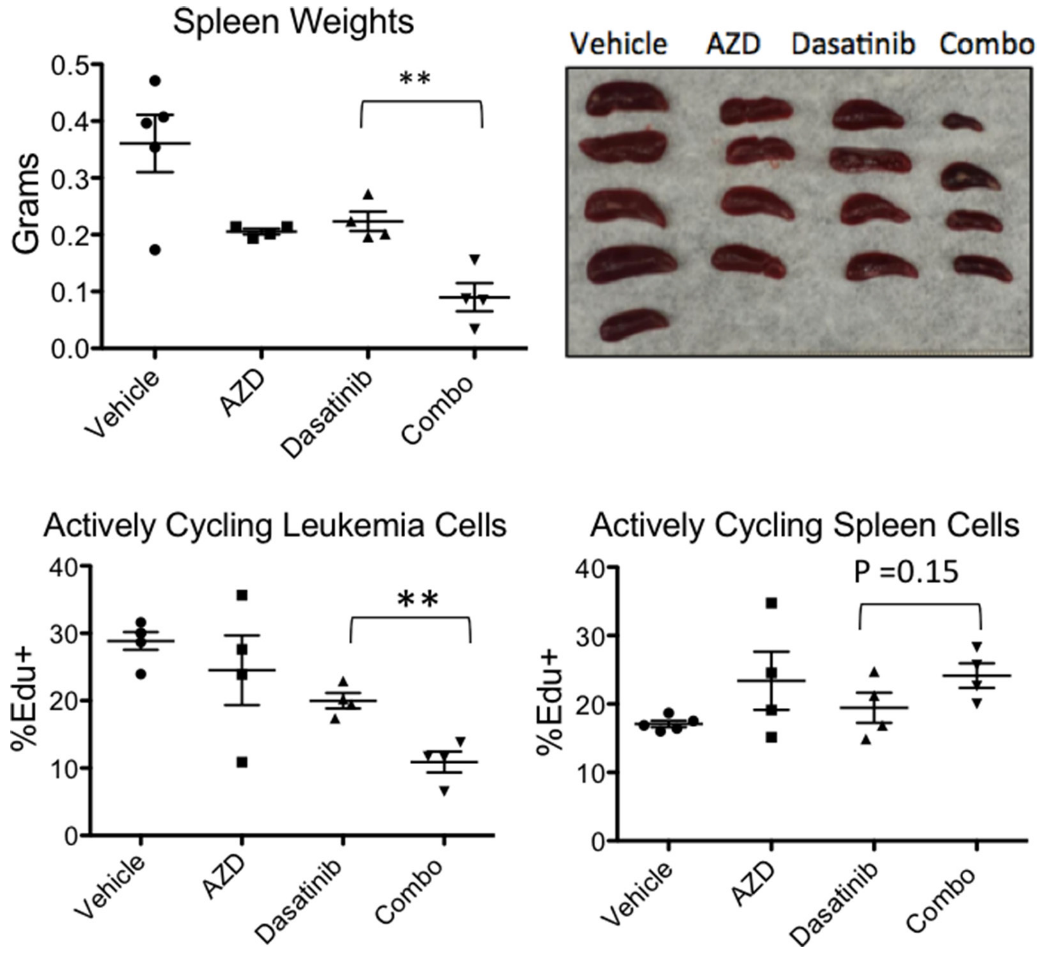

Supplementary Figure 1: PDX model of Ph-like B-ALL (PAUXZX) treated with dasatinib (2.5 mg/kg) or AZD8055 (20 mg/kg) or combination for 5 days. Decreased spleen size in combination group as compared to either single treatment group and significantly decrease in spleen weight in the combination group when compared to dasatinib group ($p = 0.006$). Actively cycling cells were identified by the incorporation of Edu in leukemia cells and endogenous bone marrow cells. There was a significant decrease in actively cycling leukemia cells identified by hCD19 in the combination group as compared to the dasatinib group ($p = 0.01$) and preserved actively cycling endogenous bone marrow cells as identified by mCD45.

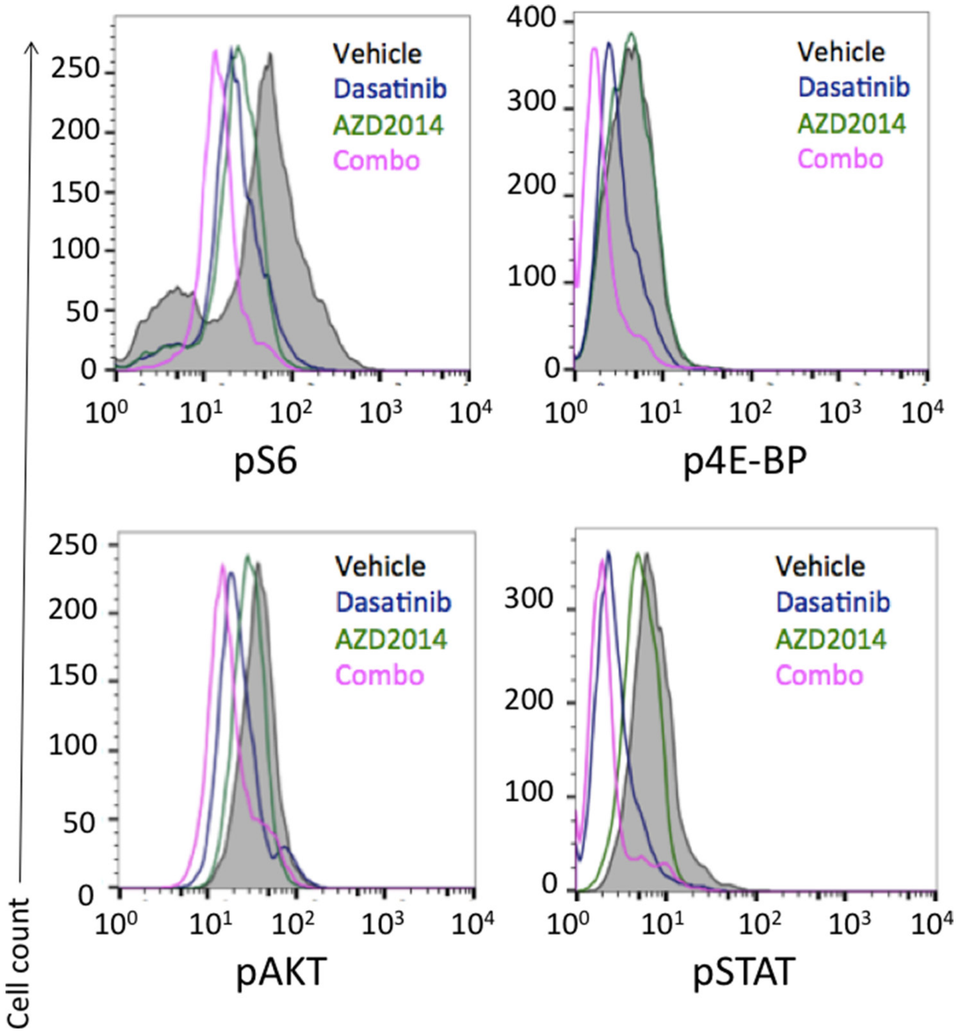

Supplementary Figure 2: Representative histograms from phosphoflow analysis of individual mice presented in Figure 2.

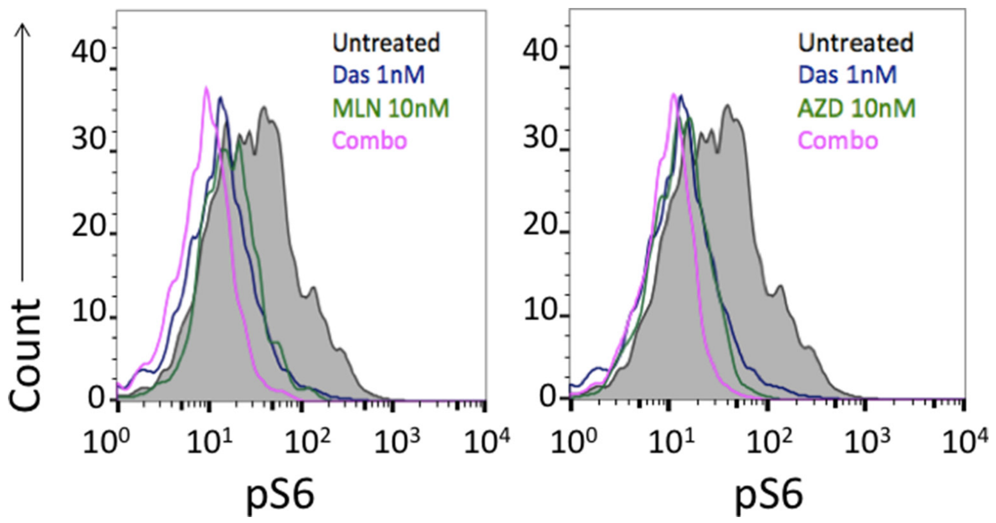

Supplementary Figure 3: Representative histograms from p-S6 phosphoflow analysis of leukemia cells from *ETV6-ABL1* ALL PDX model cultured *in vitro* on stroma.

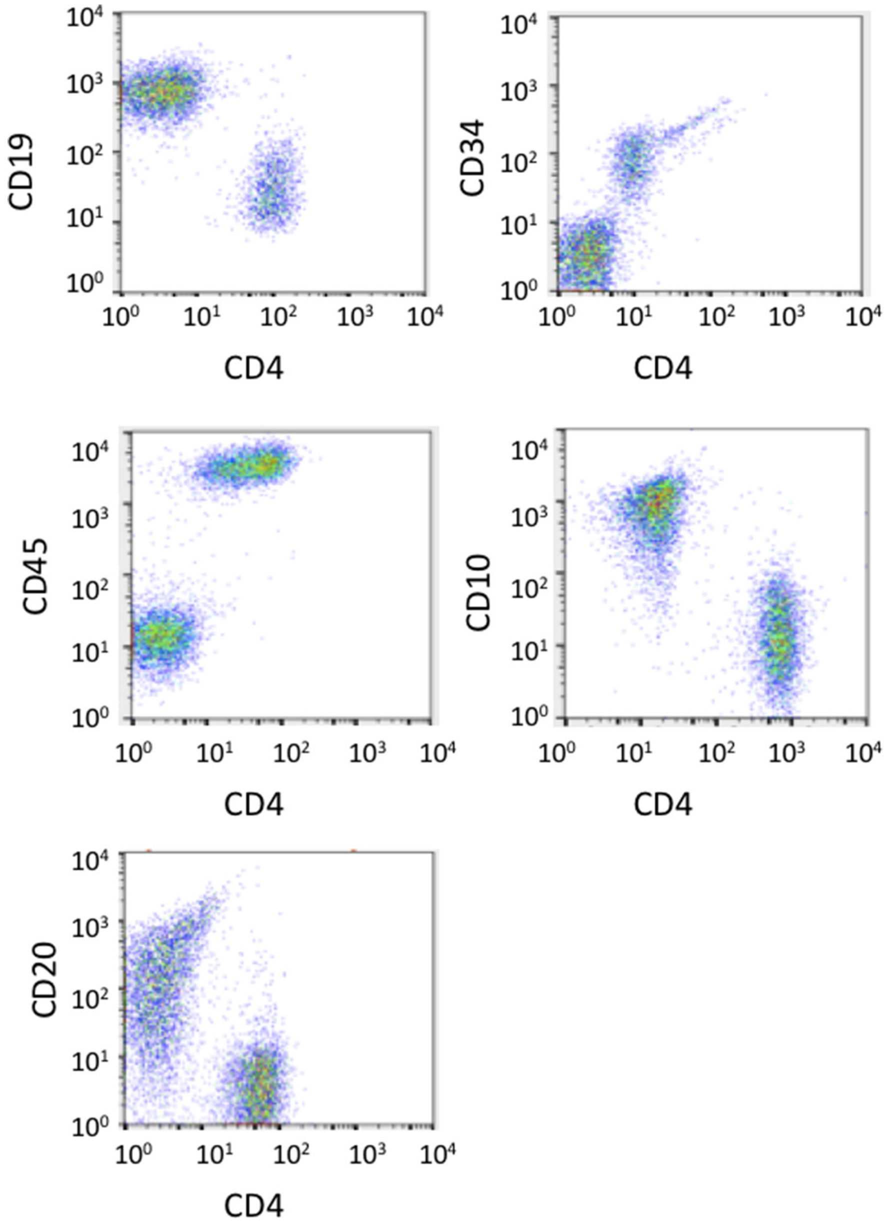

Supplementary Figure 4: Ph-like ALL TVA1 cell line immunophenotyping. AML3 is human CD4+, CD19–, CD10− and CD34– AML cell line that was used as a negative control for B-ALL markers.16 Flow cytometry was used to identify the following markers expressed on TVA1 cells: CD19, CD20, CD10, and markers absent (CD34) or low (CD45).
